# Transcriptome Sequencing and Analysis of Leaf Tissue of *Avicennia marina* Using the Illumina Platform

**DOI:** 10.1371/journal.pone.0108785

**Published:** 2014-09-29

**Authors:** Jianzi Huang, Xiang Lu, Wanke Zhang, Rongfeng Huang, Shouyi Chen, Yizhi Zheng

**Affiliations:** 1 College of Life Science, Shenzhen Key Laboratory of Microbial Genetic Engineering, Shenzhen University, Shenzhen, China; 2 Institute of Genetics and Developmental Biology, Chinese Academy of Sciences, Beijing, China; 3 Biotechnology Research Institute, Chinese Academy of Agricultural Sciences, Beijing, China; Institute of Genetics and Developmental Biology, Chinese Academy of Sciences, China

## Abstract

*Avicennia marina* is a widely distributed mangrove species that thrives in high-salinity habitats. It plays a significant role in supporting coastal ecosystem and holds unique potential for studying molecular mechanisms underlying ecological adaptation. Despite and sometimes because of its numerous merits, this species is facing increasing pressure of exploitation and deforestation. Both study on adaptation mechanisms and conservation efforts necessitate more genomic resources for *A. marina*. In this study, we used Illumina sequencing of an *A. marina* foliar cDNA library to generate a transcriptome dataset for gene and marker discovery. We obtained 40 million high-quality reads and assembled them into 91,125 unigenes with a mean length of 463 bp. These unigenes covered most of the publicly available *A. marina* Sanger ESTs and greatly extended the repertoire of transcripts for this species. A total of 54,497 and 32,637 unigenes were annotated based on homology to sequences in the NCBI non-redundant and the Swiss-prot protein databases, respectively. Both Gene Ontology (GO) analysis and Kyoto Encyclopedia of Genes and Genomes (KEGG) pathway analysis revealed some transcriptomic signatures of stress adaptation for this halophytic species. We also detected an extraordinary amount of transcripts derived from fungal endophytes and demonstrated the utility of transcriptome sequencing in surveying endophyte diversity without isolating them out of plant tissues. Additionally, we identified 3,423 candidate simple sequence repeats (SSRs) from 3,141 unigenes with a density of one SSR locus every 8.25 kb sequence. Our transcriptomic data will provide valuable resources for ecological, genetic and evolutionary studies in *A. marina*.

## Introduction

*Avicennia marina*, commonly known as grey mangrove, is an ecologically important tree species with a widespread distribution throughout the Indo-West Pacific between the latitudes of 25°N and 38°S [1]. The earliest fossil pollen of *A. marina* has been found in Australia [2]. This species belongs to the family of Acanthaceae and is a foundation species of the mangrove forest ecosystem, which plays an essential role in supporting biodiversity, maintaining coastal stability, and protecting water quality [3]. In the coastal societies, *A.marina* has long been utilized as a source of wood, tannins, resins, fodder, or medicines [4]. Despite its ecological and sociological importance, *A. marina* has been increasingly threatened by various human activities and insect pests [5], [6]. Efficient strategies are, therefore, urgently needed for the conservation and sustainable management of this species.

As a pioneer tree, *A. marina* usually occurs at the seaward edge of mangrove forests where salinities are close to full-strength seawater. To survive in such high-salinity conditions, *A. marina* has developed a series of adaptive characteristics, such as salt exclusion by root ultrafiltration, selective uptake and transport of ions, salt secretion via foliar glands, and accumulation of compatible solutes [7]. In addition to these morphological and physiological traits, the tolerance of *A. marina* to the high-salinity environment is also tightly linked to the regulation of gene expression, which makes this halophytic species an ideal candidate for learning molecular mechanisms underlying ecological adaptation and exploring genes conferring increased tolerance to salt stress for crop improvement. To date, only a few salt stress-related genes have been isolated and characterized from *A. marina* [8]–[14]. Only one of them, a Cu-Zn superoxide dismutase gene (*AmSod1*), has been successfully transformed into rice, resulting in transgenic plants with improved tolerance to salt stress [15].

At the genomic level, while the whole genome sequencing is difficult or impractical for this woody plant species with 2n = 36 chromosomes [16], an EST sequencing project based on randomly selected clones from a 0.5M NaCl-stressed leaf cDNA library has generated 1,602 ESTs [17], which constitute the majority of the 1,893 ESTs currently available for *A. marina* in the NCBI database. Compared to the enormous amounts of publicly released EST data for other halophytic species, such as salt cress (*Thellungiella halophila*) and Burma mangrove (*Bruguiera gymnorrhiza*) [18], [19], the EST collections for *A. marina* are relatively scarce. Moreover, these ESTs of *A. marina* have been mainly produced through the Sanger sequencing of cDNA library, which usually suffers from an overrepresentation of preferentially cloned sequences or an inadequate representation of less-abundant transcripts [20], [21]. The paucity of EST data and the limitations of traditional EST sequencing method have greatly hindered further progress towards a better understanding of the genetic basis of ecological adaptation for *A. marina*.

In terms of molecular markers, several studies have used random amplified polymorphic DNA (RAPD), restriction fragment length polymorphism (RFLP), amplified fragment length polymorphism (AFLP), and simple sequence repeat (SSR) to evaluate the genetic variation of the extant *A. marina* populations [22]–[28]. However, these studies have employed relatively small number of genetic loci owing to the difficulty in marker development. For instance, there are now only 26 SSR loci available for *A. marina*, which have been developed by two research groups using the conventional library-based methods [24], [28]. Some of these loci show too low polymorphism to be sufficient for investigating the genetic structure of edge populations [28]. To increase the precision and accuracy of the estimates of population genetic parameters, more numbers of genetic loci are required for *A. marina*. While the sequence-based searching can fulfill rapid identification of large numbers of SSR loci with reduced costs, its application in *A. marina* is again limited by a lack of genomic or transcriptomic sequences.

Transcriptome sequencing using next generation technologies have been increasingly carried out in non-model plants for gene discovery and marker development [29], [30]. Within the mangrove community, four species have been subjected to transcriptome sequencing on either the Roche 454 GS FLX platform or the Illumina GA platform [31]–[33]. A recent study also performed high-throughput sequencing to create an inventory of the small RNAs in *A. marina* [34]. In the present study, we constructed a leaf transcriptome of *A. marina* using the Illumina sequencing platform. We described the functional characterization of this transcriptome and discussed its possible relatedness with the ecological adaptation of this halophytic species. We also presented an abundance of EST-SSR markers for the assessment of genetic variation that would contribute to devising reforestation schemes and breeding programs in *A. marina*.

## Materials and Methods

### Ethics statement

*A. marina* is widely distributed in tropical coastal regions and is not included into any list of endangered or protected species. All necessary permissions for sample collections were obtained from the Guangdong Neilingding Futian National Nature Reserve Administration.

### Plant material and RNA preparation

Leaves of *A. marina* were sampled from healthy plants grown in the Futian National Nature Reserve in Shenzhen, China. A total of 30 leaves were randomly collected from different parts of three individual plants (five young leaves and five mature leaves per plant) growing in the intertidal zone. These parts were at least 1.0 m above the high tide level. The leaves were transported to the laboratory in sterile bags, rinsed thoroughly in running water to remove the dust, surface-sterilized with 1% sodium hypochloride (NaOCl) and 75% ethanol solution, and processed within 24 hours after sampling. The leaves from each plant were separately used for RNA extraction. The total RNA was isolated using a modified CTAB method and further purified with the RNeasy Plant Mini Kit (Qiagen). RNA quality was checked with the use of a 2100 Bioanalyzer RNA Nanochip (Agilent Technologies). Equal amounts of total RNA from each of the three plants were pooled together for the following sequencing procedures.

### Sequencing and assembly

Illumina sequencing was performed at the Beijing Genomics Institute (BGI) in Shenzhen, China. Briefly, mRNAs were purified from 20 ug total RNA using oligo(dT)-attached magnetic beads and fragmented into small pieces (100–400 bp). The cleaved RNA fragments were then primed with random hexamers and submitted to the synthesis of the first-strand and second-strand cDNAs. The synthesized cDNAs were ligated with paired-end adaptors. Then the cDNA fragments with 200 bp (±25 bp) size were selected by agarose gel electrophoresis and enriched by PCR amplification. The cDNA library with 200 bp insertion fragments was constructed for sequencing on an Illumina GA IIx. Each sequencing pass could yield two 90-bp independent reads from either end of a DNA fragment.

After sequencing, the raw sequence data were first purified by trimming adapter sequences and removing low-quality sequences. The resulting clean reads were assembled using the SOAPdenovo program [35]. The clean reads were split into smaller pieces, the K-mers, and were conjoined into contigs using the *de Bruijn* graph. After a trial of different K-mer sizes, 29-mer was chosen for this study to maximize the N50 value and the amount of assembled reads in the final assembly. Subsequently, the contigs from the same transcript were identified by paired-end reads, which also helped to detect the distances between these contigs. SOAPdenovo connected contigs from the same transcript to form scaffolds, using N to represent unknown sequences between the two contigs. Then the gap fillings were carried out using the pair-end information again to retrieve read pairs with one read well-aligned on the contigs and another read located in the gap region. The resulting scaffolds with least Ns were defined as unigenes. To measure the gene abundance, the RPKM (Reads Per Kilobase per Million reads) values were calculated using SOAP2 with a maximum of three mismatches [36]. To analyze the quality of the assembly, we also downloaded the 1,893 Sanger ESTs available for *A. marina* from the NCBI database (accessed on 23 October 2013) and aligned them against the unigene dataset with a BLAST E-value cutoff of 1.0e-10. This Transcriptome Shotgun Assembly project has been deposited at DDBJ/EMBL/GenBank under the accession GBIO00000000. The version described in this paper is the first version, GBIO01000000.

### Sequence annotation

The sequence orientations of the unigenes were determined by BLASTx against the NCBI non-redundant (Nr) protein database, the Swiss-Prot protein database, the Kyoto Encyclopedia of Genes and Genomes (KEGG) pathway database, and the Cluster of Orthologous Groups (COG) database. The incongruent results from different databases were settled under a priority order of Nr, Swiss-Prot, KEGG, and COG. For the rest unigenes that were unaligned to the above databases, ESTScan was used to predict their CDS and sequence orientations [37].

For assignments of gene descriptions, the unigenes were searched against the Nr database using BLASTx with an E-value cutoff of 1.0e-5. The BLAST results were parsed by a Perl script (available on request). The results of only the best hit were extracted. Based on their Nr annotations, the unigenes were assigned Gene Ontology (GO) annotations using Blast2GO [38], followed by functional classification using the WEGO software [39]. These processes were done separately for the unigenes with Nr annotations from plant proteins and for those annotated from fungal proteins. Besides, the putative metabolic pathways for the unigenes were assigned by performing BLASTx against the KEGG pathway database with the E-value cutoff of 1.0e-5.

### SSRs identification

The perl script program MISA (MIcroSAtellite; http://pgrc.ipk-gatersleben.de/misa/) was used to detect and locate SSRs in the unigenes. The searching criteria were set as follows: at least six contiguous repeats for di-, five repeats for tri- and tetra-, and four repeats for penta- and hexa-nucleotide motifs. Considering the difficulties of distinguishing genuine mononucleotide repeats from polyadenylation products or single-nucleotide stretch errors generated by sequencing, this type of repeats were excluded from the searching process. The maximal number of bases interrupting two SSRs in a compound microsatellite was 100. We determined which SSRs occurred in coding regions of genes by extracting the aligned portions of sequences having BLAST matches to annotated protein-coding orthologs in the Nr database, and then used the same algorithm as above to detect SSRs in both the aligned and remaining portions of these unigenes.

## Results

### Illumina sequencing and *de novo* assembly

Using Illumina sequencing technology, we generated a leaf transcriptome with approximately 4 GB of raw data. After removal of low-quality reads and adaptor-containing reads, 40 million clean reads remained. The Q20 percentage (sequencing error rate <1%) and overall GC percentage of these clean reads were 91.20% and 47.35%, respectively. All sequence data have been deposited in NCBI Sequence Read Archive (SRA) under the accession number SRA065788. Based on the clean reads, 642,221 contigs were assembled with a mean length of 143 bp (Table S1). With the help of paired-end reads, contigs derived from the same transcript were identified and the distances between these contigs were inferred. This contig-joining procedure yielded 151,384 scaffolds with a mean length of 338 bp (Table S1). The scaffolds were further processed by a gap-filling step, which resulted in 91,125 unigenes with a mean length of 463 bp and a total length of about 42.2 Mb (Table 1). The length of these unigenes ranged from 200 to 5,125 bp, and there were 26,149 unigenes (28.70%) more than 500 bp long (Table S1).

**Table 1:** Statistics for the unigenes of *Avicennia marina*.

Category	Value
Total number of unigenes	91,125
Mean length of unigenes (bp)	463
N50 of unigenes (bp)	528
Total length of unigenes (bp)	42,187,266
Average number of reads per unigene	154
Average coverage (rpkm)	22.5
Number of unigenes with Nr annotations	54,497
Number of unigenes with Swiss-Prot annotations	32,637
Number of unigenes with GO annotations	11,472
Number of unigenes with COG annotations	15,852
Number of unigenes with KEGG annotations	23,966

To evaluate the quality of transcriptome assembly, we realigned all the usable sequencing reads to the unigenes. The average number of reads per unigene is 154 (Table 1), suggesting that the unigenes were well overlapped by the sequencing reads. The RPKM values of these unigenes ranged from 0 to 1,309 (Table S2). The random distribution of reads in the unigenes showed that the unigenes were evenly covered by the reads with relatively fewer reads in the 3′ ends of them (Figure S1). The gap distribution of the unigenes indicated that the majority of them displayed a ratio of gap length to gene length less than 5% (Figure S2). The assembling results were further assessed by comparing unigenes to the publicly available Sanger ESTs of *A. marina*. Among the 1,893 Sanger ESTs, 1,755 (92.71%) had strong BLAST hits to 3,480 unigenes (Table S3). On average, 71.52% of the length of these Sanger sequences was aligned to at least one unigene sequences. The average number of aligned unigenes for each Sanger EST sequence was 3.28 and the average alignment length were 220 nucleotides. The alignments between Sanger and unigene sequences were 85.38% identical for all alignments and 94.23% for each Sanger sequence's best BLAST alignment.

To reduce possible artifacts generated during assembling process, we removed the assembled transcripts with less than 1× coverage, which resulted in 88,271 unigenes for further analyses. By using reciprocal BLAST analysis with an E-value cutoff of 1.0e-5 and an aligned-length cutoff of 100 bp, we identified 38,475 unigenes with high similarity to at least one of the other unigenes in this transcriptome.

### Sequence annotation

**Figure 1:**
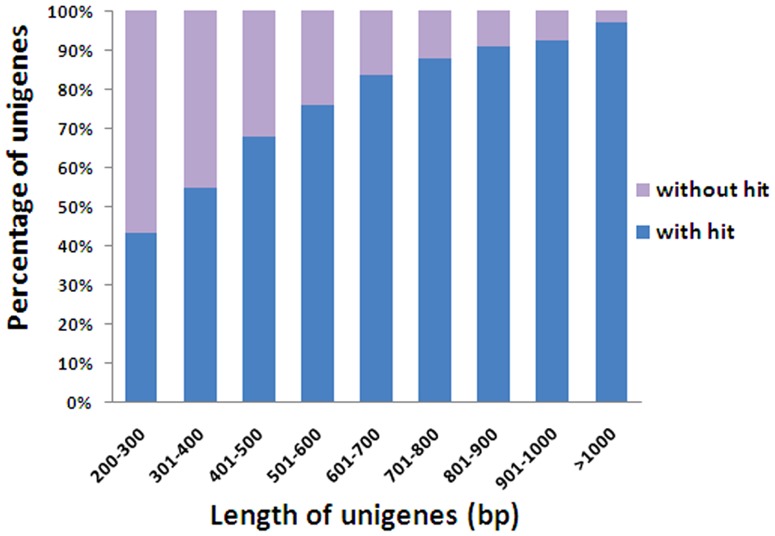
Comparison of the length distribution of unigenes with or without hits.

To understand the functions of the *A. marina* unigenes generated in this study, we compared these sequences with those in public databases. A BLASTx of these 88,271 unigenes with an E-value threshold of 1.0e-5 against the Nr protein database and Swiss-Prot protein database generated 54,497 (61.74%) and 32,637 (36.97%) matches, respectively (Table 1). Only 43.79% of the unigenes shorter than 300 bp had significant BLAST hits in the Nr database. In contrast, the proportion of unigenes with hits increased sharply to 97.11% for those with a size over 1,000 bp (Fig. 1). The length distribution of unigenes with Swiss-Prot annotations showed a similar tendency. In other words, the longer unigenes were more likely to have BLAST matches in the protein databases. Totally, BLAST searches identified 30,679 unique protein accessions from the Nr databases, indicating that our sequencing project had generated a substantial number of genes for *A. marina*.

**Figure 2:**
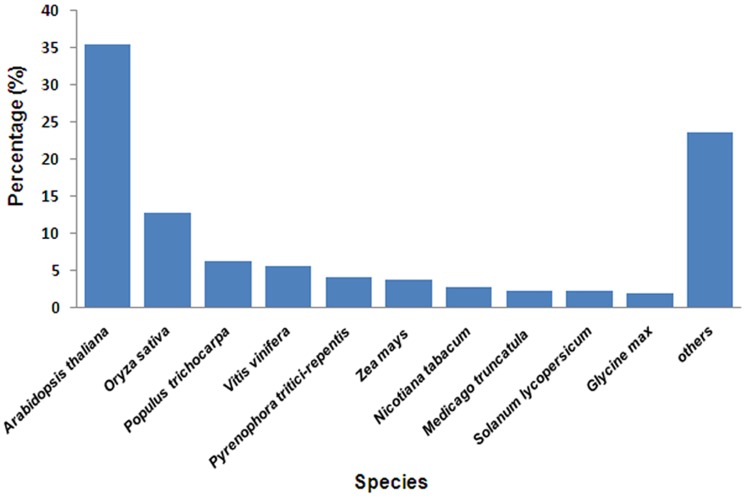
Species distribution of Nr annotation results.

Among the 54,497 unigenes with Nr annotations, 49,344 (90.54%) had top-hit BLAST result from plants (Table 2). Besides, a considerable portion of unigenes received an annotation from fungi (4,874, 8.94%), mainly with species from Ascomycetes and Basidiomycetes. The remaining less than one percent of unigenes were annotated by protein accessions from algae (262, 0.48%) and other eukaryotes (17, 0.03%). About 76% of the annotated unigenes were matched with the sequences from the top ten species, i.e., *Arabidopsis thaliana* (19,316, 35.44%), *Oryza sativa* (6,886, 12.64%), *Populus trichocarpa* (3,347, 6.14%), *Vitis vinifera* (3,029, 5.56%), *Pyrenophora tritici-repentis* (2,232, 4.10%), *Zea mays* (2,025, 3.72%), *Nicotiana tabacum* (1,459, 2.68%), *Medicago truncatula* (1,175, 2.16%), *Solanum lycopersicum* (1,153, 2.12%), and *Glycine max* (997, 1.83%) (Fig. 2). Among these ten species, *N. tabacum* and *S. lycopersicum* from the plant order of Solanales are closely related to *A. marina* from Lamiales according to the Angiosperm Phylogeny Group III (APG III) system [40]. *Py. tritici-repentis* is a fungal species [41]. The other seven plant species all have available genome sequences generated by completed large-scale sequencing projects. There were also 77 unigenes with top hits from *A. marina* protein accessions in the Nr database, which had been characterized by a handful of previous studies.

**Table 2:** Annotation Sources for *Avicennia marina* unigenes.

Annotation sources	Number of matched unigenes
Plants	49,344
Fungi	4,874
-Ascomycetes	4,525
*–Pyrenophora*	2,232
*–Phaeosphaeria*	544
*–Penicillium*	341
*–Aspergillus*	277
*–Ajellomyces*	180
-Basidiomycetes	276
-other fungi	73
Algae	262
Other eukaryotes	17

For the 4,874 unigenes annotated with fungal sequences (denoted as fungus-derived unigenes hereafter), *Pyrenophora* (2,232), *Phaeosphaeria* (544), *Penicillium* (341), *Aspergillus* (277), and *Ajellomyces* (180) were the five genera with the most abundant matches (Table 2). Totally, the top hits for these fungus-derived unigenes came from 184 fungal species (Table S4). Compared with the whole transcriptome set, this subset contained a larger proportion of unigenes shorter than 500 bp and a less proportion of unigenes longer than 500 bp. As a result, the mean length of this subset of unigenes was much shorter than that of the unigenes annotated with plant sequences (denoted as plant-derived unigenes hereafter) (Table 3). Furthermore, the fungus-derived unigenes were mapped by less sequencing reads and had lower coverage than the plant-derived unigenes. Taken together, these results suggested that the leaf transcriptome not only comprised plenty of plant transcripts, but also contained low-abundance transcripts from a wide range of fungi.

**Table 3:** Comparison of the characteristics of plant-derived and fungus-derived unigenes.

Item	Plant-derived unigenes	Fungus-derived unigenes
Mean length of unigenes (bp)	572	405
Average number of reads per unigene	224	44
Average coverage (rpkm)	29.5	6.1
Number of unigenes with GO annotations	10,040	1,352
Number of SSR-containing unigenes	1,583	98

### GO classification and KEGG analysis

**Figure 3:**
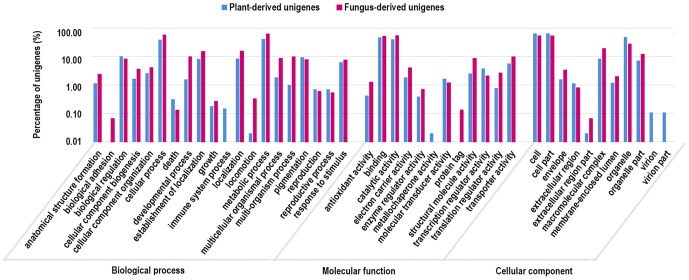
Gene Ontology classifications of plant-derived and fungus-derived unigenes.

Following their Nr annotations, we retrieved GO annotations for 11,472 unigenes from the GO database (Table 1). The remaining unigenes with Nr annotations failed to obtain a GO term, largely due to their uninformative descriptions (e.g., unknown, putative, or hypothetical proteins). 10,040 out of 49,344 (20.35%) plant-derived unigenes were assigned 28,259 GO terms in the three main categories, including 5,435 unigenes with terms from ‘Biological Process’, 6,932 unigenes from ‘Molecular Function’, and 6,445 unigenes from ‘Cellular Component’ (Fig. 3). Among them, 3,053 unigenes had an assignment in all three categories. In ‘Biological Process’ category, the two most abundantly represented sub-categories were metabolic process (4,063, 40.47%) and cellular process (3,875, 38.59%), followed by biological regulation (971, 9.67%), pigmentation (896, 8.92%), localization (814, 8.11%), and response to stimulus (582, 5.79%). In ‘Molecular Function’ category, most of the unigenes were functionally correlated with binding (4,373, 43.56%) and catalytic activity (3,804, 37.89%), followed by transporter activity (555, 5.53%) and transcription regulator activity (348, 3.47%). As for ‘Cellular Component’ category, most of the unigenes were located in cell part (6,402, 63.76%), while only a few unigenes were sorted into virion part (11, 0.11%) or extracellular region part (2, 0.02%).

Likewise, 1,352 out of 4,874 (27.74%) fungus-derived unigenes were assigned 4,560 GO terms (Table 3) and they also covered a broad range of GO categories (Fig. 3). Nevertheless, there were some minor differences in the distribution of GO categories between these fungus-derived unigenes and those plant-derived ones. For example, in ‘Biological Process’, the fungus-derived unigenes were apparently more highly represented in developmental process, multicellular organismal process, multi-organism process, and locomotion than the plant-derived unigenes, whereas the former set of unigenes were completely absent in immune system process. It was also noteworthy that both plant- and fungus-derived unigenes were highly represented in biological regulation, localization, pigmentation, and response to stimulus. In ‘Molecular Function’, the fungus-derived unigenes were less represented in molecular transducer activity and transcription regulator activity, but they were more represented in all the other types of activity in comparison to the plant-derived unigenes. In ‘Cellular Component’, the fungus-derived unigenes were more highly represented in envelope, macromolecular complex, membrane-enclosed lumen and organelle part.

**Figure 4:**
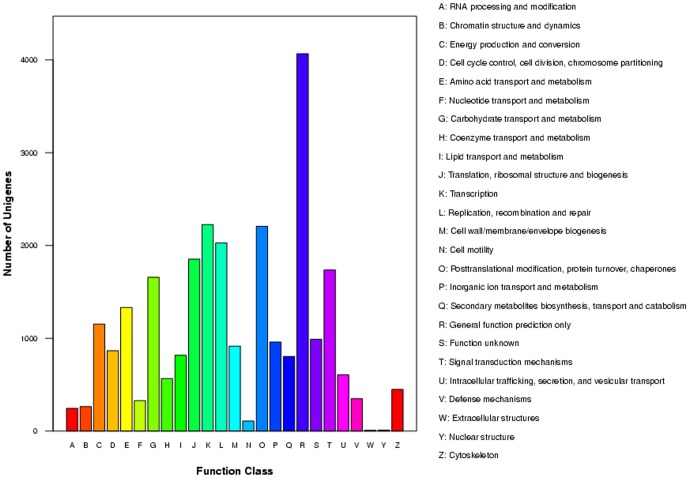
Clusters of orthologous group classification.

For further functional prediction and classification, all unigenes were subjected to a search against the COG database. 15,852 out of 88,271 (17.96%) unigenes were assigned into 25 COG categories with 26,516 functional terms (Fig. 4). ‘General function prediction only’ (4,064, 15.33%) was found to be the major category, followed by ‘Transcription’ (2,224, 8.39%), ‘Posttranslational modification, protein turnover, chaperones’ (2,206, 8.32%), ‘Replication, recombination and repair’ (2,027, 7.64%), ‘Translation, ribosomal structure and biogenesis’ (1,852, 6.98%), ‘Signal transduction mechanisms’ (1,735, 6.54%), ‘Carbohydrate transport and metabolism’ (1,657, 6.25%), ‘Amino acid transport and metabolism’ (1,330, 5.02%), and ‘Energy production and conversion’ (1,152, 4.34%). Only a few unigenes were classified into ‘Nuclear structure’ (10, 0.04%) and ‘Extracellular structures’ (8, 0.03%).

Lastly, we performed the KEGG pathway analysis to identify the biological pathways active in the leaf of *A. marina*. As a whole, 23,966 out of 88,271 (27.15%) unigenes were assigned 134 KEGG pathways (Table 1). The number of unigenes in each pathway ranged from 3 to 5,949 (Table S5). In addition to the metabolic pathways, which always ranked the first place in pathway-based analysis, biosynthesis of secondary metabolites, plant-pathogen interaction, spliceosome and ribosome also appeared in the top 10 pathways most represented by the *A. marina* unigenes (Table 4). Besides, a large number of unigenes were involved in the microbial metabolism in diverse environments.

**Table 4:** The top 10 KEGG pathways with the highest numbers of *Avicennia marina* unigenes.

Rank	Pathway	Number of unigenes*	Pathway ID
1	Metabolic pathways	5949 (24.82%)	ko01100
2	Biosynthesis of secondary metabolites	3103 (12.95%)	ko01110
3	Microbial metabolism in diverse environments	1492 (6.23%)	ko01120
4	Plant-pathogen interaction	1377 (5.75%)	ko04626
5	Spliceosome	977 (4.08%)	ko03040
6	Carbon metabolism	881 (3.68%)	ko01200
7	Ribosome	828 (3.45%)	ko03010
8	Biosynthesis of amino acids	796 (3.32%)	ko01230
9	Starch and sucrose metabolism	637 (2.66%)	ko00500
10	Protein processing in endoplasmic reticulum	614 (2.56%)	ko04141

*Percentages of unigenes in each pathway among 23,966 unigenes with KEGG annotations are shown in parentheses.

### Characterization of SSR markers

SSRs are valuable markers for conservation and breeding programs. We analyzed the 88,271 unigenes generated in this study to mine EST-based SSR markers. A total of 3,423 SSRs were found in 3,141 unigenes, including 2,886 and 255 unigenes with one and more than one SSR, respectively (Table 5). 198 SSRs were present in compound formation. On average, one SSR could be found every 8.25 kb in the unigenes. Among these SSR-containing unigenes, there were 1,583 plant-derived and 98 fungus-derived unigenes (Table 3). The rest were unannotated unigenes. The density of SSRs was much lower in coding regions (one EST-SSR per 36.81 kb) than in non-coding regions (one EST-SSR per 6.89 kb). Meanwhile, this density was much lower in coding regions from plant-derived unigenes (one EST-SSR per 39.32 kb) than in those from fungus-derived unigenes (one EST-SSR per 23.79 kb). Of the identified repeats, 2,418 (70.64%) had sufficient flanking sequence to allow for PCR primer design (Table S6).

**Table 5:** Summary of SSRs identified from the *Avicennia marina* transcriptome.

Searching item	Number
Total number of unigenes examined	88,271
Total number of identified SSRs	3,423
Number of SSR containing unigenes	3,141
Number of unigenes containing more than one SSR	255
Number of SSRs present in compound formation	198
Di-nucleotide repeats	2,214
Tri-nucleotide repeats	952
Tetra-nucleotide repeats	75
Penta-nucleotide repeats	63
Hexa-nucleotide repeats	119

**Figure 5:**
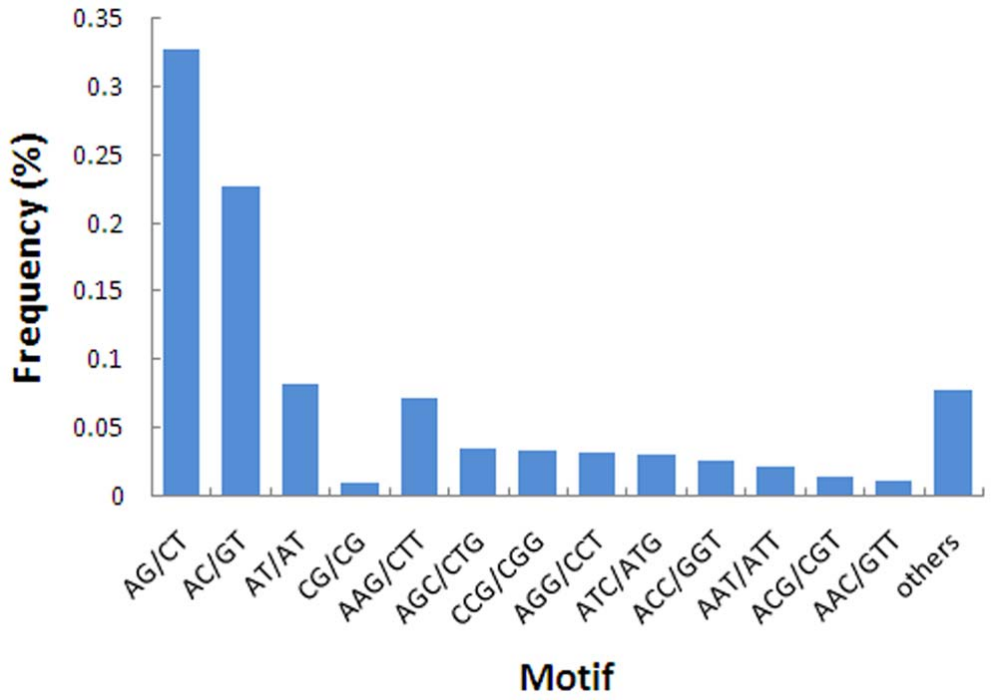
Frequency distribution of SSRs based on motif sequence types.

The major types of the identified SSRs were di-nucleotide (2,214, 64.68%) and tri-nucleotide (952, 27.81%) (Table 5). Within these SSRs, 138 motif sequence types were identified, of which di-, tri-, tetra-, penta-, and hexa-nucleotide repeats had 4, 10, 19, 31 and 74 types, respective. The most frequently occurring type of di-nucleotide repeat was AG/CT (1,120, 32.72%), followed by AC/GT (776, 22.67%), AT/AT (284, 8.30%) and GC/CG (34, 0.99%). Within the tri-nucleotide repeats, AAG/CTT (248, 7.25%) was the most common type, followed by AGC/CTG (118, 3.45%), CCG/CGG (114, 3.33%), AGG/CCT (112, 3.27%) and ATC/ATG (106, 3.10%). The remaining 129 types of motifs accounted for 14.96% in total (Fig. 5). The majority of these EST-SSRs (3,399, 99.30%) were 12 to 30 bp. There were only 24 EST-SSRs with length longer than 30 bp (Table S7).

## Discussion

Transcriptome characterization using next generation sequencing (NGS) technologies has proven reliable and effective for study on stress adaptations in plants [31], [42]. Mangroves live under complex environmental conditions featured by flooding, hypoxia, strong wind, high temperatures, and high salinities, which offer them unique advantages in research on plant adaptation to abiotic stress. Here, we reported the first NGS-based transcriptome sequencing and analysis of a most salt-tolerant mangrove species, *A. marina*, for which sequence data is very limited in the public database at present. Illumina sequencing and *de novo* assembly resulted in 91,125 unigenes with a mean length of 463 bp. 61.74% of the *A. marina* unigenes had BLAST matches against the Nr database. This percentage of unigenes with Nr annotations was higher than those reported in *R. mangle* and *H. littoralis* (44.12%), and in *M. pinnata* (50.27%) [31], [33].

In this transcriptome, there was an excess of highly similar unigenes (38,475 out of 88,271 unigenes), which likely resulted from recently duplicated paralogs, alternatively spliced transcripts, or variant alleles of the same gene. Gene duplications could lead to constitutively higher gene expression and were proposed to play a significant role in shaping genomes for stress adaptations [43], [44]. Alternative splicing also acted as an important regulatory mechanism in plant responses to external stimuli, and was particularly prevalent in plants exposed to environmental stress [45], [46]. Therefore, to some extent, the excess of unigenes in *A. marina* transcriptome might be associated with its stress adaptation. Besides, since the natural *A. marina* populations were found to be generally outcrossing with little or no inbreeding [47], there would inevitably be a substantial amount of variant alleles in this leaf transcriptome. Lastly, the excess of unigenes might also result from unjoined contigs representing different regions of the same gene.

Within the annotated unigenes, 77 had BLAST matches with *A. marina* protein accessions, including several previously studied salt-responsive genes. The first characterized salt-responsive gene in *A. marina* was a chloroplast-localized *BADH* gene, which was mainly expressed in leaf and significantly induced at high salinity [8]. In our study, unigene43463 matched a partial CDS of the *AmBADH* gene. Three betaine/proline transporters, i.e. *AmT1*, *AmT2* and *AmT3*, also appeared to be involved in the accumulation of betaine under high salinity conditions [9]. Our study showed that *AmT1* was matched by three unigenes (unigene638, 62646 and 72942) corresponding to different regions with a little overlap in the same gene. Likewise, *AmT2* and *AmT3* were matched by three (unigene2578, 17508 and 46860) and four unigenes (unigene28938, 32247, 64000 and 82708), respectively. *AmFer1* was a ferritin gene that displayed a short-term induction in response to salt stress [10]. This gene was also matched by three overlapping unigenes (unigene14963, 66863 and 70353). *Am-pAPX1* encoded a peroxisomal ascorbate peroxidase and was observed to gradually up-regulate under salt stress [12]. Our results indicated that two unigenes (unigene70202 and 70894) had the best hits with separate regions of *Am-pAPX1*. A dehydrin gene was also shown to be activated by salinity in leaves and roots of *A. marina* [13], and it was matched by unigene54734. Taken together, the above examples demonstrated that the transcriptome of *A. marina* held immense potential for screening out candidate stress-related genes.

A notable feature of this leaf transcriptome was the existence of an extraordinary high proportion of unigenes (8.94%) with BLAST hits to fungal proteins. Comparatively, in the transcriptome of *M. pinnata*, only less than one percentage of unigenes had best hits to non-plant sequences [33]. Considering that our leaf samples were collected from different parts of multiple healthy trees and strictly surface-sterilized before RNA extraction, the above observation should not be caused by sampling bias, plant disease, or exogenous contamination. On the other hand, previous studies have already documented the occurrence of foliar endophytes in *A. marina* and some other mangrove species [48]–[50]. Hence, those unigenes annotated with fungal proteins were most likely to represent the massive transcripts from endophytic fungi in *A. marina*.

The fungal endophytes are a group of microorganisms that reside inside plant tissues, usually not causing any disease symptoms. Historically, the research on fungal endophytes mainly focused on the isolation of bioactive substances with potential medical, agricultural and industrial applications. During the past decade, there have been increasing interests in the ecological significance of fungal endophytes as they were noticed to be able to confer biotic and abiotic stress tolerance to their host plants [51], [52]. The common strategy for elucidating the mechanisms of plant-fungal interactions depends on the isolation, culture and identification of fungal endophytes. However, a complete collection of all fungal endophytes in a host is hardly accessible, given that these fungi often exist in complex communities rather than individual organisms and most of them are unculturable using conventional microbiological techniques.

In this study, we exemplified the utility of NGS-based transcriptome sequencing and annotation in surveying fungal diversity as a whole without isolating them one by one from plant tissues. We detected the presence of transcripts annotated by sequences from 178 fungal species, which were much more than the species identified after isolation from leaf tissues of *A. marina* [48], [49]. Consistent with previous findings, Ascomycetes and Basidiomycetes were the two most common taxonomic groups of endophytic fungi in *A. marina*. For the five genera with the most abundant matches, *Phaeosphaeria*, *Penicillium* and *Aspergillus* have already been recorded in *A. marina* [48], [49], [53]. *Pyrenophora* and *Ajellomyces* were recorded in *A. marina* for the first time and both genera have abundant genomic sequences in public databases. Our results should have overestimated the real number of fungal species owing to the inherent nature of annotation process. That is, transcripts from the same species may be annotated by sequences from different species. Nevertheless, it would be undoubted that the transcripts in this transcriptome had revealed some formerly unidentified species of fungal endophytes in *A. marina*.

According to their Nr annotations, 10,040 plant-derived unigenes and 1,352 fungus-derived unigenes were assigned GO annotations. As in all the plant transcriptomes reported so far, metabolic process and cellular process in ‘Biological Process’, as well as binding and catalytic activity in ‘Molecular Function’, were the most predominant categories in this transcriptome. Further, the plant-derived unigenes in this transcriptome showed a high representation in the transporter activity category. This bias towards transporter in GO profiles was evident in all halophytic species and supposed to be critical to salt stress tolerance [54]. The GO profiles of fungus-derived unigenes were generally in accordance with those of plant-derived unigenes, with the exception that the former ones were much more highly represented in developmental process, multicellular organismal process, multi-organism process, and locomotion. These processes might be closely related to the colonization of fungi in plant tissues. Our results demonstrated that NGS-based metatranscriptomic analysis could simultaneously investigate the transcriptional landscapes of both host plant and its endophytes.

KEGG analysis revealed that 23,966 unigenes were involved in 134 pathways. Similar to other plant transcriptomes, the categories of metabolic pathways, spliceosome and ribosome were well represented in this transcriptome. Additionally, there were large quantities of unigenes responsible for the biosynthesis of secondary metabolites. These secondary metabolites, produced by plant, fungi or both, might represent chemical adaptations to environmental stresses. For example, the accumulation of phenylpropanoids in plants has been observed under pathogen attack, wounding, high light, low temperature, low nitrogen, or low phosphate conditions, and it was proposed as a defense strategy against biotic or abiotic stress [55]. The overrepresentation of transcripts in phenylpropanoid biosynthesis pathways was also noticed in *M. pinnata* transcriptome [33]. A recent study has also described a similar increase of triterpenoid content with salt concentration in salt-secreting *A. marina* and non-secreting *Rhizophora stylosa* [56]. The abundance of unigenes involved in microbial metabolism and plant-pathogen interaction again supported the existence of diverse fungal endophytes in *A. marina*.

To our knowledge, only 26 SSR loci have been developed for genetic diversity analysis of *A. marina* through the library-based methods [24], [28]. In the current study, we identified 3,423 candidate SSRs from 3,141 out of 88,271 *A. marina* unigenes. Both the frequency (3.56%) and the density (one SSR per 8.25 kb) of SSRs in this transcriptome are relatively low comparing with the range of frequencies and densities reported for other dicotyledonous species [57]. There were much more di-nucleotide SSRs than tri-nucleotide SSRs. The relative abundance of di- and tri-nucleotide SSRs varied among different studies in light of genome composition, the sizes of dataset, and the searching parameters [58]. Although these SSRs developed from transcriptomic sequences are less polymorphic, they have higher cross-species transferability and are tightly linked with functional genes that may control important traits.

In conclusion, we constructed a leaf transcriptome of *A. marina* with a large number of unigenes using the Illumina sequencing technology. The dataset developed in this study greatly extended the repertoire of transcripts for *A. marina*, which would contribute to the discovery of stress-responsive genes for functional study and further application in crop improvement. We detected an extraordinary amount of transcripts derived from fungal endophytes and demonstrated for the first time the utility of transcriptome sequencing in surveying species diversity of endophytes, which were also ecologically important but hard to isolate and culture out of plant tissues. In addition, we identified an abundance of gene-linked SSR markers from the transcriptomic dataset and designed relative primers for PCR amplifications. These candidate SSR markers, though not tested in the wet-lab yet, would provide valuable resources for future ecological and evolutionary studies in *A. marina*.

## Supporting Information

Figure S1 **Random distribution of reads in** ***Avicennia marina*** **unigenes.** The x-axis indicates the relative position of sequencing reads in the unigenes. The orientation of unigene is from 5′ end to 3′ end.
(TIF)

Figure S2 **Gap distribution of** ***Avicennia marina*** **unigenes.** The gap distribution represents the percentage of the number of N divided by the sequence length of unigenes.
(TIF)

Table S1 **Length distribution of contigs, scaffolds and unigenes.**
(XLSX)

Table S2 **The RPKM values for 91,125** ***Avicennia marina*** **unigenes.**
(XLSX)

Table S3 **Summary for BLAST of** ***Avicennia marina*** **Sanger EST sequences against unigenes.**
(XLSX)

Table S4 **Numbers of** ***Avicennia marina*** **unigenes with hits in 184 fungal species.**
(XLSX)

Table S5 **Numbers of** ***Avicennia marina*** **unigenes in 134 KEGG pathways.**
(XLSX)

Table S6 **PCR primers and information for 2418 SSRs identified from** ***Avicennia marina*** **transcriptome.**
(XLSX)

Table S7 **Length distribution of EST-SSRs based on the number of repeat units.**
(XLSX)
