# Supplementary figures and images for: Transcriptome Sequencing and Analysis of Leaf Tissue of Avicennia marina Using the Illumina Platform
Source: PLoS One. 2014 Sep 29;9(9):e108785. doi: 10.1371/journal.pone.0108785 (PMC4181315; doi:10.1371/journal.pone.0108785)

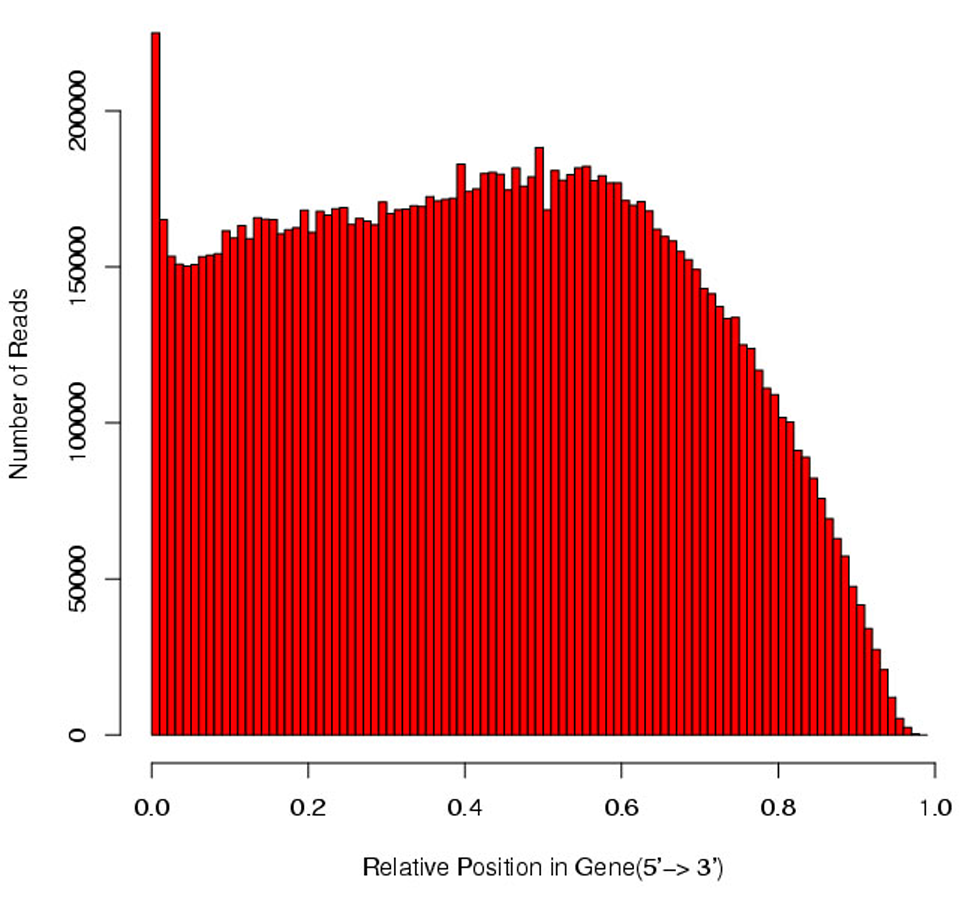

Supplement: Figure S1 — Random distribution of reads in Avicennia marina unigenes. The x-axis indicates the relative position of sequencing reads in the unigenes. The orientation of unigene is from 5′ end to 3′ end. (TIF) [file pone.0108785.s001.tif]

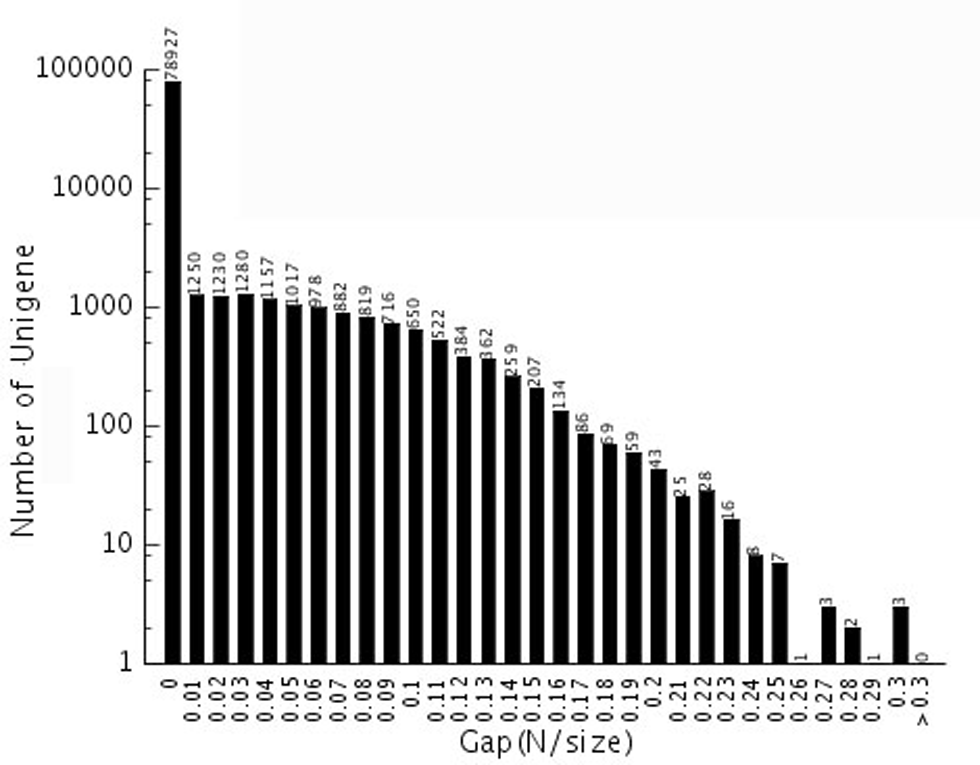

Supplement: Figure S2 — Gap distribution of Avicennia marina unigenes. The gap distribution represents the percentage of the number of N divided by the sequence length of unigenes. (TIF) [file pone.0108785.s002.tif]
